# Editorial to the Special Issue “MicroRNA Dysregulation in Tumor Occurrence, Progression and Response to Therapy”

**DOI:** 10.3390/ijms22042156

**Published:** 2021-02-22

**Authors:** Marilena V. Iorio

**Affiliations:** Research Department, Fondazione IRCCS Istituto Nazionale dei Tumori, 20133 Milan, Italy; marilena.iorio@istitutotumori.mi.it

In the last 20 years, the involvement of microRNAs in the biology of human tumors has been clearly demonstrated, and the scientific community has switched from an initial skepticism to an increasing interest toward what was called the “dark side” of DNA. Indeed, it is known that ncRNAs were initially thought to be some sort of genome waste, an idea fostered by the dogma that the DNA could pass information downstream only through a coding-protein mRNA. Instead, a huge amount of crucial information was hidden through the bases of unknown noncoding RNA strings.

Aberrant microRNA expression in neoplastic cells, microenvironment components and body fluids such as blood has been associated with disease diagnosis, prognosis and responsiveness to therapies in different tumor types, both solid tumors and hematological malignancies [1].

The special issue includes six papers, five reviews and one original paper, exploring the biological role of microRNAs and ncRNAs in different tumor types and the potential clinical applications. A crucial role of microRNAs has been described here in rhabdomyosarcoma development [2], in chronic lymphocytic leukemia [3] and in breast cancer, with a specific focus on miR-205 [4].

miRNAs are involved not only in the biology of cancer, in the occurrence and progression of the disease, but also in mechanisms of resistance to different antineoplastic therapies. For instance, miRNAs can promote responsiveness to radiation therapy by inhibiting DNA repair mechanisms, thus suggesting the possibility to exploit them as radio-sensitizers to minimize the side effects in hepatocellular carcinoma patients [5].

It is also well known that miRNAs can be produced by different cell types and released into the circulation, and circulating miRNAs (ct-miRNAs) have been described as promising noninvasive, stable and easy-handling biomarkers. Dr. Di Cosimo and colleagues [6] analyzed plasma samples from HER2-positive breast cancer patients receiving trastuzumab-based neoadjuvant treatment within the NeoALTTO trial to identify changes in ct-miRNAs levels during the first two weeks of treatment and their association with response to treatment and outcome.

ct-miRNAs can be found free in the circulation or encapsulated in exosomes. MicroRNAs can indeed be exploited as cellular messages and transferred from one cell type to another encapsulated in extracellular vesicles (EVs) [7]. EV-associated miRs act as autocrine, paracrine and endocrine factors, participating in cancer pathogenesis by modulating intercellular communication.

The topics described in this special issue support the potential use of both tissue and ct-miRNAs as biomarkers and even as tools to interfere with biological pathways crucial for tumor survival and progression. Even though there still is a long path ahead before a concrete clinical application, the research is heading in that direction.
